# The Cholera outbreak in Karachi, Pakistan: Challenges, efforts and recommendations

**DOI:** 10.1016/j.amsu.2022.103873

**Published:** 2022-05-31

**Authors:** Hira Anas Khan, Waniyah Masood, Amna Siddiqui, Shkaib Ahmad, Yumna Salman, Mohammad Yasir Essar

**Affiliations:** a Faculty of Medicine, Dow Medical College, Dow University of Health Sciences, Pakistan; b Karachi Medical and Dental College, Karachi City, Pakistan; c D.G Khan Medical College, DG Khan, Pakistan; d Afghanistan National Charity Organization for Special Diseases (ANCOSD), Kabul, Afghanistan; e Kabul University of Medical Sciences, Kabul, Afghanistan

**Keywords:** Cholera, Healthcare, Pakistan

## Abstract

Sindh Health Authorities have declared a new outbreak of cholera in Karachi, Pakistan after 129 lab-confirmed cases were reported from the Central, East, and South districts. With COVID-19 already having wreaked havoc on the country's health condition in the past years, any neglect in early preventative measures against this cholera outbreak implies progression to a perilous situation with millions of individuals at stake of acquiring the disease. Factors contributing to the occurrence of cholera outbreak include poor hygiene practices, overpopulation, increasing poverty and climate change. Appropriate responsive approaches by health authorities in cooperation with the World Health Organization (WHO) must be implemented to address the situation accordingly.

## 1 Introduction

According to the World Health Organization, cholera is an acute diarrheal infection caused by the ingestion of the bacterium *Vibrio Cholerae (V.Cholerae)* via contaminated food and water [1].

*V.Cholerae*, the gram-negative bacterium, has many serotypes which are differentiated according to its O-antigen. O1 and O139 variants cause most cases. The O1 serogroup is further subclassified into El Tor and Classical [2]. (2) Although O139 caused catastrophic outbreaks in the 1990s, El Tor remains dominant worldwide, especially Yemen, The Democratic Republic of Congo, India, and Afghanistan [3,4].

Most exposed people are asymptomatic but can become a source of transmission as they shed V.Cholera in their stools for the first 7–14 days. However, notably, diarrhea and vomiting are the first symptoms to appear. Diarrhea has a distinct milky appearance resembling water in which rice has been rinsed. These symptoms can quickly result in fluid loss leading to dehydration, this can lead to electrolyte imbalances in the body which present as cramps and in severe cases, shock [5].

Some risk factors increase one's susceptibility to contracting cholera. Major factors include poor sanitation and contaminated water, which are more common in crowded areas affected by natural disasters. Various environmental factors such as low flow rivers, warm air temperature and heavy rainfall [6] also play an important role in cholera epidemics*. V.cholera* cannot survive in acidic environment therefore stomach serves as a defense against bacteria. In contrast, people on antacids, H-2 blockers or proton pump inhibitors lack this defensive environment and are much more susceptible to the infection [5]. People with blood group O are more prone to infection [7].

Many outbreaks have previously been reported in Pakistan. In 1881, an outbreak was reported in Lahore central Jail where out of a total of 127 cases reported, 90 people had died [8]. A cholera epidemic hit the Swat state in 1937, attacking 202 and killing 100 in total [9]. In July 2002 and June 2003, 69 cholera cases were reported by a surveillance team in a village outside of Karachi [10].

Recently, in 2022, a surge in cholera cases has been reported in Karachi. Dr. Asif Saeed, an official of the Sindh health department, who is responsible for the surveillance of infectious diseases, confirmed 129 cases of cholera which were reported from six public and private hospitals. Moreover, he gave a breakdown of these cases, stating 5 cases were reported in January 2022, 14 in February, 54 in March and 56 in the month of April [11].

## 2 Challenges

Cholera has remained a notifiable endemic disease in Pakistan ^12^with the major burden of cases being reported from densely populated, urban cities such as Karachi. The recurrence of cholera outbreaks is owed to an array of factors such as poor sanitation, hygiene practices, overpopulation with increasing poverty and inadequate infrastructure, as well as climate change due to increasing pollution [12].

The root of many administrative shortcomings in Karachi is the flawed population count. As opposed to the 2017 census results which cap the numbers at approximately 16 million, the actual population is believed to be around 35 million [13]. This discrepancy automatically affects the allocation of funds for development, so the infrastructure no longer meets requirements. Inadequate housing, resulting in overcrowding, increases the risk of people-to-people contact transmission of cholera.

Millions of people reside in more than 600 slums in Karachi [14] with unsanitary practices, poor personal hygiene, and indiscriminate waste disposal [15]. According to a study 80% of transmission occurs via household contact [16]. Even though handwashing with soap can help curb cholera transmission significantly in such settings [17], such people often do not have resources or motivation for it.

91% of Karachi's water supply is unfit for drinking, with high contamination with E coli, Shigella in addition to Cholera [18]. According to a study, only 38% of water samples were free from coliforms after boiling and filtering [19]. Noncompliance with such protocol due to a lack of awareness and accessibility to required resources removes even this minor protection for a majority. Additionally, increasing poverty means regular purchase of safe, bottled water is not a sustainable alternative for most people. Food crops grown with contaminated water also result in transmission of cholera.

An estimated poverty rate of 8.9% [20] results in a drastic malnourishment crisis for millions in Karachi. This especially affects children under 5, as worsened immunity and wasting results in high child mortality rates due to infectious diseases especially diarrhea and pneumonia [21]. Therefore, with the impact of covid 19 and the recent surge in measles cases with 1861 cases reported from September 2021 to February 2022 [22], cholera might cause significant complications in such children. It is also important to note that the city's healthcare system is unequipped to deal with the large burden of outbreaks.

The recurrence of cholera outbreaks also has a strong association with climatic processes, especially after natural disasters such as earthquakes, floods, and droughts [23]. In coherence with this finding, a similar rise in diarrheal diseases and other infections was seen after record breaking rainfall in Karachi in 2020 [24] due to cross contamination and inadequate sewage treatment [25]. With minimal improvements being made to tackle such conditions, the unpredictable nature of weather conditions and subsequent disasters can lead to a greater frequency of such outbreaks.

However, the severity of the situation is also underestimated due to the lack of proper surveillance and reporting. From 1993 to 2005, Pakistan reported 0 cases of cholera to WHO [12]. While cases do come to attention, large numbers may be potentially overlooked especially in rural areas without access to proper healthcare, making it difficult to plan localized strategies. Owing to the lack of awareness and education in such settings, transmission of cholera is rampant since patients might not take precautions to protect others around them from infection. Furthermore, insufficient follow-up of cases runs the risk of emergence of drug-resistant strains which might increase the recurrence of life-threatening outbreaks.

## 3 Efforts

In 2010, the Ministry of Health in Pakistan and World Health Organization (WHO) responded to a similar cholera outbreak by expanding the post-disaster Disease Early Warning Systems (DEWS) with the primary objective of early detection and monitoring of epidemic outbreaks [26]. Alternatively, 22 US aid groups raised 10.6 million dollars to curb the disastrous impacts of flooding, ensure the accessibility of clean drinking water, and to cope with the cholera surge [27]. Other interventions include the installation of more than 60 diarrheal treatment centers across 46 flood affected districts of Pakistan [28].

Following the current cholera outbreak in Karachi, Sindh Health Authorities has taken responsive measures with the support of WHO country representatives to strengthen the surveillance system for cholera. These measures include the establishment of separate wards at tertiary care hospitals (for the suspected and laboratory confirmed cases of the cholera to prevent the contact with non-infected patients), providing 200,000 water purifying tablets to citizens in affected districts of Karachi (to prevent any further disease transmission via water resources), and an increase in laboratory diagnostic testing (enhancing early detection and rapid response strategies against the disease). Besides this, they have also initiated community engagement programs, hiring 60 social mobilizers, and introducing awareness sessions aimed at addressing information regarding the disease and its prevention [29].

Other response measures taken by Sindh Health Authorities are routinely reporting the suspected, confirmed cases and any deaths due to cholera as per the guidelines of International Health Regulations (IHR) 2005 to international health authorities [29]. In addition, Karachi Water and Sewerage Board is directed to take extra precautions while chlorinating the water and reducing the contamination score to ensure a safer water supply [11]. Besides this, health and food safety departments are also requested to maintain proper sanitation and hygiene via analyzing and monitoring the food samples [29].

## 4 Recommendations

The Cholera cases reported so far in the city are masquerading as it is not an outbreak [11]. Therefore, a strategy based on risk-informed preparedness before it turns into an epidemic must be conceptualized to combat cholera effectively in the city of Karachi, which has limited resources to execute emergency responses.

For starters, actions must be taken to strengthen the surveillance system including improvements in laboratory analysis and data management, to keep a justified track of Cholera cases, especially in high-risk towns in the city [30]. This will contribute to efficiently managing the surge in Cholera cases by promoting early detection, followed by prompt management [1].

Training the general public via workshops and campaigns to manage Cholera-related diarrhea effectively can aid in lessening complications, thereby reducing the burden on hospitals, and eventually reducing mortality. Furthermore, implementing Water, Sanitation and Hygiene (WASH) to effectively control and prevent Cholera transmission [31]. Moreover, implementation of awareness campaigns aimed at personal hygiene promotion, household water treatment and disinfection, network chlorination and safe sewage disposal. The application of Oral Cholera Vaccines (OCV) can be significantly effective in reducing the incidence of Cholera cases [32]. Lessons learnt while dealing with the COVID-19 pandemic can also have a profound effect on strategizing for eliminating the risk of Cholera outbreak in the city. As the former answered, the most cost-effective ways of vaccine or resources mobilization with methods to enhance the uptake and sustainability of response measures. It has also taught how to engage meaningfully with at-risk communities in designing and implementing interventions [33].

Since rural areas lack resource mobilization and awareness, they demand extra effort in order to curb cholera transmission. Cholera prevention in this part of the population can be made possible by creating vibrant and informative resources such as e-posters and eye-catching visual documentaries with short and easy to understand awareness messages in their respective languages. Further, these should not only demonstrate the WASH program, but also motivate people by portraying the effectiveness of OCV and routine implementation of WASH activities in lowering the chances of cholera. Additionally, airing them on different platforms of communication that are mostly used, i.e., radio stations, WhatsApp etc. will contribute more to spreading useful management tips.

Furthermore, lifesaving progress in cholera research should be promoted, primarily focusing on analyzing the effectiveness of diagnostic tests and developing improved vaccine regimens to bolster the connection between emergency response and long-term prevention [34].

Conclusively, executing the aforementioned recommendations would make it easier to tackle this all-too-precedented disease from affecting vulnerable populations.

## Ethical approval

N/A.

## Sources of funding

None.

## Authors’ contributions

All authors equally contributed.

## Consent

N/A.

## Registration of research studies

N/A.

Name of the registry:

Unique Identifying number or registration ID:

Hyperlink to your specific registration (must be publicly accessible and will be checked):

## Guarantor

N/A.

## Declaration of competing interest

None.
